# A Case of Adentulous Neuralgia

**Published:** 1872-06

**Authors:** 


### ARTICLE VI.

### *A Case of Adentulous Neuralgia.*

Geo. L., æt. 78 years, came to the clinic complaining of an agonizing pain in the body of the lower jaw, and limited to the left side of the face. He had suffered from it almost constantly for the preceeding fifteen years. When he lay down at night the pain sometimes disappeared, but attacked him again early in the morning. It existed on the other side of the mouth for about ten years, then shifted to its present position. The pain was paroxysmal, without twitchings of the muscles. While describing it he suffered so as to be scarcely able to speak. He had all his teeth extracted years ago, was otherwisse healthy, and with no neuralgia in any other part of his body.

This affection was brought to the notice of the profession as a new form of neuralgia, by Professor Gross, some years ago. In a report which he then published, he collated and described a large number of cases both clinical and private, together with his deductions as regarded the pathology of the disease. He found that adentulous subjects were liable to a form of neuralgia caused by bony matter or fibrous deposit encroaching upon the nerve fibres in the alveolar process. After extraction of the teeth the gum changes in shape and character. The alveoli are absorbed and the gum becomes so hard as to be serviceable in masticating food. The nervous filaments may be compressed by this indurated condition, or by the periosteum in the dental canal, so that the nerve fluid is interrupted and pain produced. The pathology of pain is that the action of the nerve is interfered with at the seat of the lesion. Pain in the stomach is sometimes caused by a cramp of the muscular fibres which, by contracting upon and compressing the nerve filaments, interferes with their action. The nerve current cannot be transmitted in the usual way, and explodes at the part, producing the neuralgia.

This form of neuralgia, caused by imprisonment of nervous tissue, existed in the patient. The only way in which the affection could be cured was by trephining the body of the bone near the angle, exposing and removing half an inch of the inferior dental nerve. This operation has been performed in many cases, and always afforded relief. It will be used in the present case when he returns for operation. In the meantime

℞. Morphiæ sulphat, grs. $\frac{1}{3}$.

Sig.—Every night.

—*Med. and Surg. Reporter.*

---

